# In Vitro Conditioning of Adipose-Derived Mesenchymal Stem Cells by the Endothelial Microenvironment: Modeling Cell Responsiveness towards Non-Genetic Correction of Haemophilia A

**DOI:** 10.3390/ijms23137282

**Published:** 2022-06-30

**Authors:** Silvia Barbon, Elena Stocco, Senthilkumar Rajendran, Lorena Zardo, Veronica Macchi, Claudio Grandi, Giuseppe Tagariello, Andrea Porzionato, Paolo Radossi, Raffaele De Caro, Pier Paolo Parnigotto

**Affiliations:** 1 Section of Human Anatomy, Department of Neuroscience, University of Padova, 35121 Padova, Italy; silvia.barbon@unipd.it (S.B.); elena.stocco@unipd.it (E.S.); veronica.macchi@unipd.it (V.M.); andrea.porzionato@unipd.it (A.P.); raffaele.decaro@unipd.it (R.D.C.); 2 Foundation for Biology and Regenerative Medicine, Tissue Engineering and Signaling—TES, Onlus, 35030 Padova, Italy; claudio.grandi@unipd.it (C.G.); pierpaolo.parnigotto@unipd.it (P.P.P.); 3 Department of Surgery Oncology and Gastroenterology, University of Padova, 35124 Padova, Italy; senthilstem@gmail.com; 4 Haematology and Haemophilia Centre, Castelfranco Veneto Hospital, 31033 Castelfranco Veneto, Italy; lorena.zardo@aulss2.veneto.it (L.Z.); tagariello.giuseppe@gmail.com (G.T.)

**Keywords:** adipose-derived stem cells, Haemophilia A, coagulation factor VIII, endothelial differentiation, stem cell therapy, regenerative medicine

## Abstract

In recent decades, the use of adult multipotent stem cells has paved the way for the identification of new therapeutic approaches for the treatment of monogenic diseases such as Haemophilia A. Being already studied for regenerative purposes, adipose-derived mesenchymal stem cells (Ad-MSCs) are still poorly considered for Haemophilia A cell therapy and their capacity to produce coagulation factor VIII (FVIII) after proper stimulation and without resorting to gene transfection. In this work, Ad-MSCs were in vitro conditioned towards the endothelial lineage, considered to be responsible for coagulation factor production. The cells were cultured in an inductive medium enriched with endothelial growth factors for up to 21 days. In addition to significantly responding to the chemotactic endothelial stimuli, the cell populations started to form capillary-like structures and up-regulated the expression of specific endothelial markers (CD34, PDGFRα, VEGFR2, VE-cadherin, CD31, and vWF). A dot blot protein study detected the presence of FVIII in culture media collected from both unstimulated and stimulated Ad-MSCs. Remarkably, the activated partial thromboplastin time test demonstrated that the clot formation was accelerated, and FVIII activity was enhanced when FVIII deficient plasma was mixed with culture media from the untreated/stimulated Ad-MSCs. Overall, the collected evidence supported a possible Ad-MSC contribution to HA correction via specific stimulation by the endothelial microenvironment and without any need for gene transfection.

## 1. Introduction

Stem cell therapy research is advancing rapidly as a novel regenerative approach to treat both acquired and genetic diseases [1]. The rationale behind this is that patients suffering from organ and tissue dysfunctions can be treated with transplanted stem cells which have the potential to restore specific tissue compartments [2].

Over the last few decades, Haemophilia A (HA) has been investigated among the genetic diseases that could be treated with this regenerative strategy. There is not yet a definitive cure for this pathology, and current therapy consists of the so-called replacement treatment, which aims to sufficiently increase the concentration of the missing coagulation factor VIII (FVIII) to prevent or stop spontaneous and traumatic hemorrhages [3,4].

With the progress in biomedical engineering, preclinical and clinical research has started to conceptualize and develop long-term treatments for HA by using gene and cell therapy. Although recently receiving promising results in phase I/II trials [5,6], adeno-associated virus (AAV) gene therapy is not exempt from important concerns, including capsid-specific T-cell responses, which reduce transgene expression and cause hepatotoxicity. Another consideration to be made is in regards to the fact that viral vector production on a clinical scale is costly and variable [7]. Furthermore, the clinical development of AAV-FVIII still appears to be more complicated in comparison with AAV-factor IX (FIX) due to (1) the difficulty of efficient AAV vector packaging for large FVIII cDNA, and (2) the uncertain choice of liver target cell [8]. Notably, phase I/II studies reporting long-term follow-up highlighted an unexpected decline in FVIII levels after 1 year and continuously during the following 5 years post AAV-mediated expression [9,10].

According to experimental and clinical experience, the cell compartments that produce FVIII are mainly represented by endothelial sinusoidal cells. In fact, it is well known that liver-transplanted patients resolve the hemorrhagic disease [11,12]. Considering that, HA cell therapy is based on the hypothesis that the transplantation of exogenous cells capable of releasing FVIII could become a functional cure for coagulation deficiency. A variety of genetically modified cells has been tested so far as delivery vehicles for FVIII, including hematopoietic stem/progenitor cells [13,14,15], bone marrow-derived mesenchymal stem cells [7,16,17], endothelial cells [18], human placental cells [19], and adipose stromal cells [20]. Despite promising pre-clinical results, the development of effective cell therapy approaches for HA treatment may benefit from providing insights into non-genetically modified cell populations able to produce FVIII and endowed with high engraftment potential. Indeed, pre-clinical studies have already demonstrated the therapeutic potential of non-transduced cell populations in correcting the Haemophilia A phenotype, resorting to the transplantation into animal HA models of tissue-specific cells such as Liver Endothelial Sinusoidal Cells (LESCs) [21] and human pluripotent stem cell (hPSC)-derived LSEC progenitors [22]. Based on the previous research, either hepatocytes or LSECs could be candidate cells for the therapeutic intervention of HA; nevertheless, the transplantation of stem cells rather than already specialized cells seems to be the most promising approach due to the greater proliferative and differentiation potential of cell populations with stem/progenitor characteristics [23]. For Haemophilia A stem cell therapy, the efficacy of bone marrow-derived cell transplantation has already been demonstrated by Follenzi and collaborators [24]. Bone marrow has been considered for ages the main multipotent stem cell source for regenerative therapies, but its clinical use faces some practical difficulties, such as extraction procedures, which are highly invasive for the donor, and cell yield, which is highly variable and dependent on the age of the donor [25]. Thus, more accessible stem cell sources have recently been studied, such as peripheral blood [26,27,28] and adipose tissue [29,30]. In recent years, Adipose-derived Stromal Cells (ADSCs) have proven to have ideal characteristics for regenerative purposes [31]. In addition to decreased sampling risk for individual donors, easier method for isolation, and a higher number of isolated stromal cells, ADSCs are superior to BMSCs in some biological features, including the immunomodulatory properties and the secretion of a variety of growth factors and cytokines, as well as anti-apoptosis and anti-inflammation potential [32]. What is more, in vitro ADSC cultures are identified by an active proliferation rate, a multidifferentiative capacity for mesodermal, endodermal, and ectodermal lineages [33], and a lack of major histocompatibility class II (MHC II) [34]. This last feature appears to be fundamental for the treatment of genetic pathologies such as Haemophilia A since the patient cells are affected by the mutation. Using non-immunogenic stem cells, the isolation source can safely be allogenic without having to fall into the genetic modification of autologous cells.

This work investigated the in vitro conditioning of a commercial adipose-derived stromal cell line of human origin, called Adipose-derived Mesenchymal Stem Cells (Ad-MSCs), to in vitro model cell responsiveness towards the endothelial microenvironment, which may resemble the sinusoidal endothelial niche. Evidence has already been reported in the literature about ADSC differentiative potential towards the endothelial lineage [35,36,37], with scant studies having also preliminarily demonstrated that undifferentiated and non-transfected human adipose stem cells express FVIII [38] at low but significant levels (0.03 µg/mL per 10^5^ cells in 48 h; ~30% of normal human plasma FVIII level) [20]. However, a systematic in vitro characterization of ADSC responsiveness towards the endothelial microenvironment in terms of FVIII expression is currently missing. Thus, for the first time in ADSC literature, we herein focused on their ability to express and release FVIII after specific endothelial stimulation and without any genetic manipulation in order to make them acquire LESC-like functionality.

## 2. Results

### 2.1. Characterization of Ad-MSC Cultures

**Figure 1:**
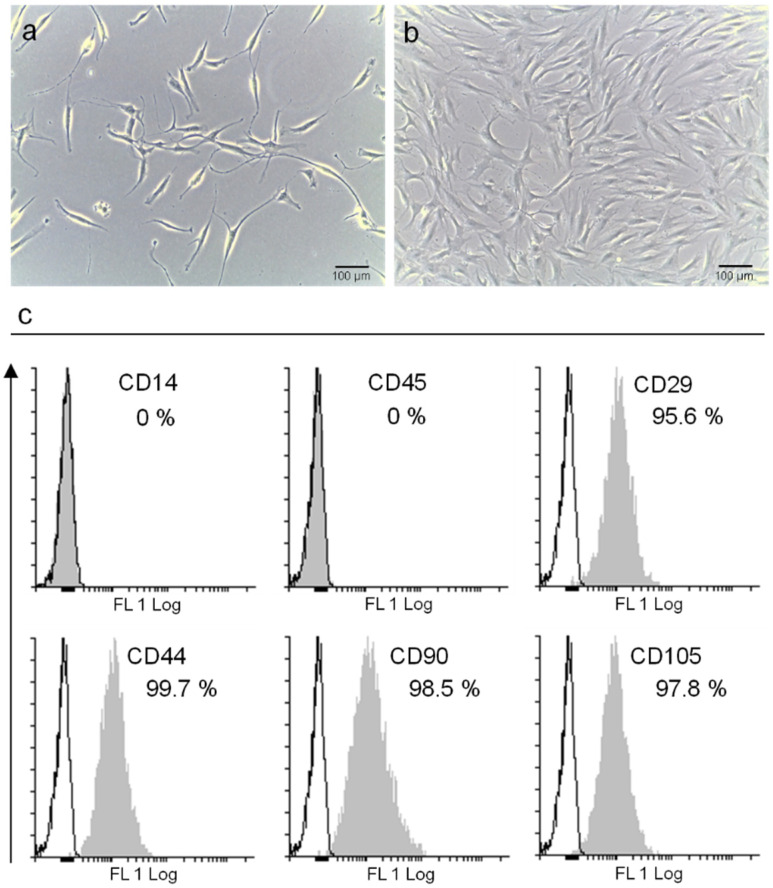
**Characterization of Ad-MSC cultures.** Morphological analysis by optical microscopy of Ad-MSCs at passage 3, in (**a**) sub-confluence and (**b**) confluence conditions (Scale bar: 100 µm). (**c**) Cytometric evaluation of Ad-MSC immunophenotype. Data are presented as percentage of positive cells (gray profile) in comparison with the isotype control (white profile).

First of all, the commercial Ad-MSC primary cell line was analyzed for its stemness features. A morphological characterization of Ad-MSC cells by optical microscopy highlighted their spindle-shaped morphology, which is typical of mesenchymal/stromal cells and was maintained over passages in sub-confluent culture conditions (Figure 1a,b). In parallel, the specific immunophenotype of Ad-MSCs was characterized by flow cytometry and defined to be CD90^+^/CD105^+^/CD44^+^/CD29^+^/CD14^−^/CD45^−^ (Figure 1c).

### 2.2. Cell Migration Capacity

**Figure 2:**
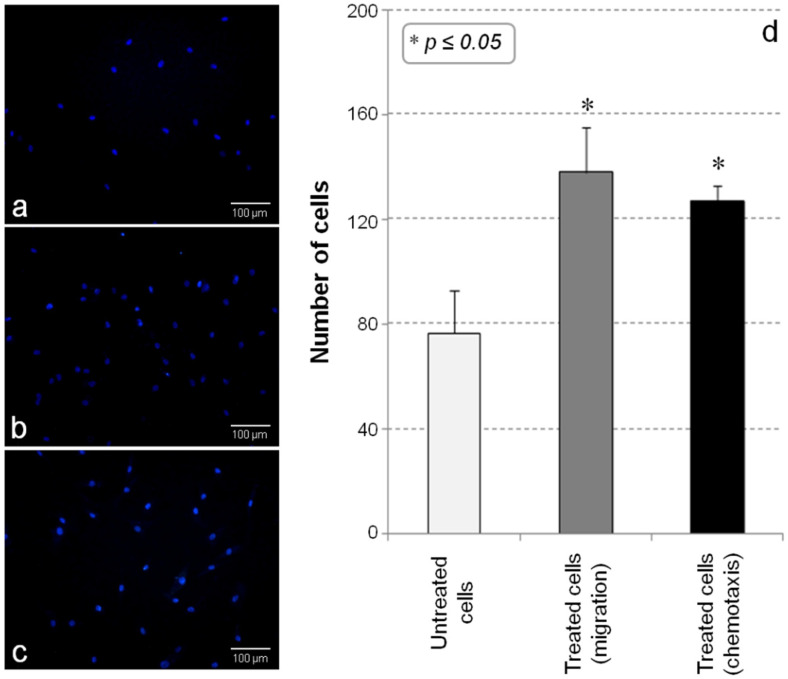
**Cell migration assay.** Optical microscopy of Ad-MSCs migrated through the porous filter after 24 h of incubation: (**a**) untreated cells vs. cultures treated with differentiation medium for (**b**) migration or (**c**) chemotaxis evaluation (Scale bar: 100 µm). (**d**) Effects on cell migration and chemotaxis of endothelial growth factors. The results are reported as mean of three different experiments ± standard deviation. (* *p* < 0.05).

The capacity of Ad-MSC to respond to the endothelial microenvironment was evaluated by testing their migration across a porous filter under the stimulation of endothelial factors [namely, vascular endothelial growth factor (VEGF), human basic fibroblast growth factor (hFGF-b), epidermal growth factor (EGF), insulin-like growth factor-1 (IGF-1), heparin, and ascorbic acid], which were present in the upper or in the bottom side of the membrane. Figure 2 shows the images of the untreated cultures (Figure 2a) and the cells treated with the endothelial medium (Figure 2b,c). The nuclei count demonstrated that Ad-MSCs are responsive to the endothelial microenvironment, as evidenced by the significant (*p* ≤ 0.05) increase in cells that crossed the filter with respect to the control cultures (Figure 2d). In particular, specific endothelial growth factors demonstrated the ability to both stimulate cell migration and exert a strong chemotactic effect on the treated cultures.

### 2.3. Endothelial Differentiation

To confirm Ad-MSC responsiveness to an endothelial-like microenvironment in vitro, the cells underwent a specific differentiation treatment through a 7-, 14- and 21-day stimulation with endothelial growth medium containing specific inductive factors, (i.e., VEGF, hFGF-b, EGF and IGF-1). In parallel, Ad-MSCs grown in a proliferation medium were considered as the undifferentiated control.

**Figure 3:**
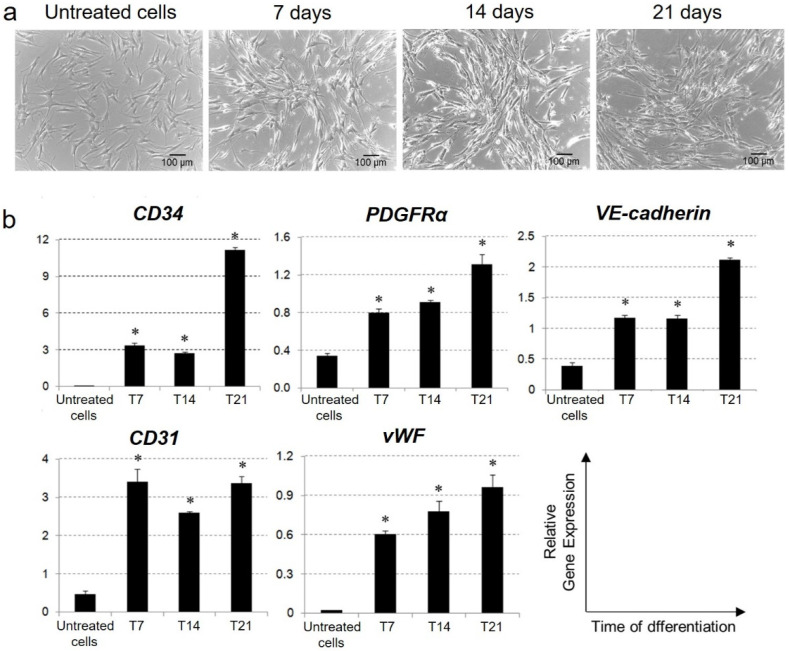
**Morphology and gene expression profile of differentiated Ad-MSCs.** (**a**) Phase-contrast micrographs of Ad-MSC population cultured in proliferative medium (untreated cells) or in endothelial differentiative medium for 7, 14 and 21 days (Scale bar: 100 µm). (**b**) Real Time PCR analysis of endothelial mRNAs expression on untreated cells or in Ad-MSCs cultured in induction medium for 7 (T7), 14 (T14) and 21 (T21) days. Relative gene expression was defined as an n-fold difference referred to the calibrator sample (HUVEC cultures); the housekeeping gene (GAPDH) expression was considered to validate the amplification efficiency of target genes. In the right bottom of the figure, the measured units on the x-axis and y-axis of other graphs are indicated. (* *p* < 0.01). **Abbreviations:** *PDGFRα*, *Platelet Derived Growth Factor Receptor Alpha*; *VE-cadherin*, *vascular endothelial cadherin*; *vWF*, *von Willebrand factor*.

This differentiation treatment was demonstrated to induce changes in Ad-MSC morphology during the first seven days of stimulation. When cultured in an endothelial medium, the cells stopped proliferating and acquired a more elongated morphology, and started to organize into capillary-like structures (Figure 3a).

Gene expression analysis revealed that the specific induction treatment significantly stimulated the transcription of genes associated with the endothelial lineage, i.e., *CD34*, *Platelet-Derived Growth Factor Receptor Alpha* (*PDGFRα*), *vascular endothelial (VE)-cadherin*, *CD31*, and *von Willebrand factor (vWF)*. This confirmed the high responsiveness of Ad-MSCs to the endothelial microenvironment in vitro (Figure 3b).

**Figure 4:**
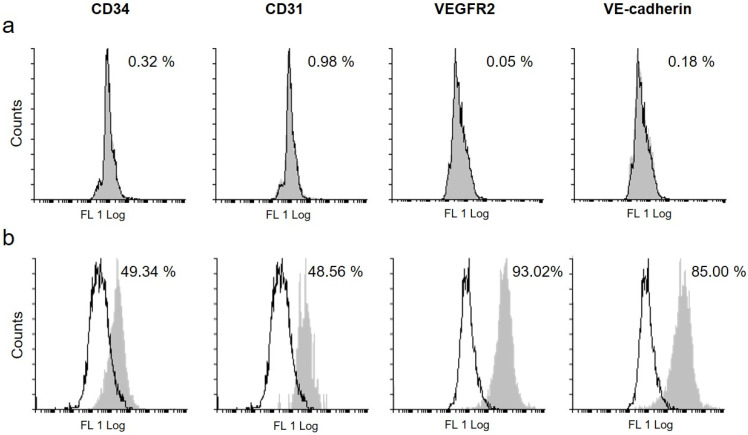
**Protein expression study.** Flow cytometry study of endothelial protein expression in (**a**) untreated cells or (**b**) Ad-MSCs cultured in induction medium for 21 days. Data are presented as percentage of positive cells (gray profile) in comparison with the isotype control (white profile). **Abbreviations:** *VEGFR2, Vascular Endothelial Growth Factor Receptor 2; VE-cadherin, vascular endothelial cadherin*.

The flow cytometry study of the differentiated Ad-MSCs and untreated controls corroborated the gene expression results, demonstrating that the stimulation with specific growth factors enhanced the expression of vascular-endothelial proteins (Figure 4). In particular, Ad-MSC cultures were found to be negative for CD31, CD34, VEGFR2, and VE-cadherin when maintained in basal conditions (Figure 4a) but were able to respond to the endothelial stimulation by activating the expression of these specific lineage proteins (Figure 4b).

### 2.4. FVIII Release

**Figure 5:**
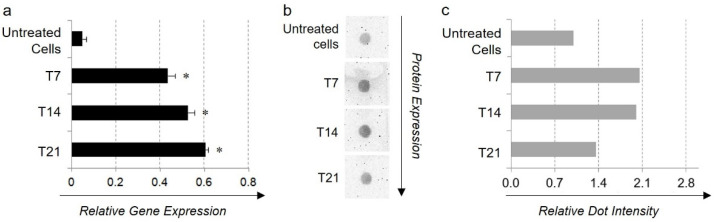
**FVIII expression.** (**a**) Real-Time PCR analysis of FVIII mRNA expression in untreated cells or in Ad-MSCs cultured in induction medium for 7 (T7), 14 (T14), and 21 (T21) days (*: *p* < 0.01 in comparison with the untreated cells). (**b**) Dot blot evaluation of FVIII release into the culture media of untreated cells and 7 (T7), 14 (T14), 21 (T21) day-differentiated Ad-MSCs. (**c**) Dot blot densitometry and relative intensity of protein dots calculated using Image J software. Results are normalized towards the undifferentiated sample.

The final aim of Ad-MSC endothelial differentiation was to verify their capacity to increase the expression and release of FVIII in response to specific stimulation treatment. Both gene (Figure 5a) and protein (Figure 5b) expression studies highlighted that Ad-MSCs expressed the FVIII gene and protein in basal conditions. When cultured in an endothelial inductive medium, they significantly up-regulated the transcription of the *FVIII* gene (*p* < 0.01) in comparison with the untreated control, whereas no significant differences were detected among the treated samples. (Figure 5a). In addition, a sustained synthesis and release of the corresponding protein over time were demonstrated following dot blot analysis on cell supernatants (Figure 5b).

### 2.5. Coagulation Assay

**Figure 6:**
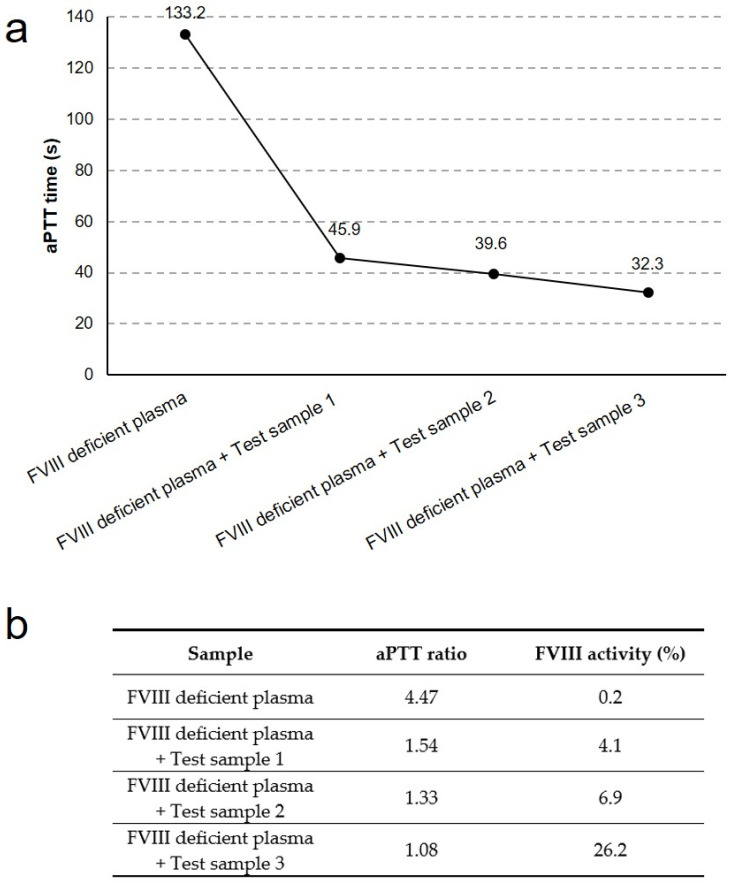
**Coagulation assay.** (**a**) aPTT reduction after mixing FVIII deficient plasma with culture medium collected from undifferentiated cells (Test sample 1) or Ad-MSCs stimulated with endothelial medium for 7 (Test sample 2), 14 (Test sample 3), and 21 days (Test sample 4). (**b**) Results of the aPTT assay reported as aPTT ratio and FVIII activity.

The activated partial thromboplastin time (aPTT) test was performed to investigate any effect of the FVIII released by untreated/differentiated Ad-MSCs on blood coagulation. Culture medium collected from undifferentiated cells (Test sample 1) or Ad-MSCs stimulated with endothelial medium for 7 (Test sample 2) and 14 days (Test sample 3) was mixed with FVIII deficient plasma, and the aPTT time was measured in seconds. The aPTT correction by this mixing suggested that the added test sample likely contained the missing coagulation factor (FVIII). In addition, the correction of the clotting time of the deficient plasma is proportional to the concentration (% activity) of FVIII in the sample (Figure 6b), interpolated from a calibration curve constructed using pooled human plasma [39]. The results of the coagulation test were also reported as the aPTT ratio (aPTT(r)) of patient-to-normal clotting time (Figure 6b), which is often used to improve the comparability of the results between different clinical laboratories [40].

As shown in Figure 6, a reduction in aPTT time and aPTT(r), together with a progressive increase in FVIII activity, were registered in supplemented vs. normal deficient plasma.

## 3. Discussion

As a monogenic disease, Haemophilia A seems to be an ideal candidate to be treated with stem cell therapy. Since the genetic mutation affects the activity of coagulation factor VIII, advanced therapies may be based on the replacement of the deficient gene with the healthy gene that generates the functional protein (gene therapy), as well as on the incorporation of a full array of wild type genes and proteins through the transplantation of healthy cells, which may be able to restore the altered ones (cell therapy) [41]. Regarding cell therapy, both pre-clinical [42,43,44] and clinical [45,46] trials have proven to successfully correct Haemophilia A phenotype by means of lentiviral and adeno-associated vector-mediated expression of FVIII in adult stem cells, autologous fibroblasts, platelets, or hematopoietic stem cells. Significant breakthroughs regarding advanced therapies for Haemophilia A are related to pre-clinical trials in the field of stem cell transplantation [21,24,47,48]. Unlike gene transfection-based strategies, the administration of non-genetically modified cells ensures to overcome the risks of DNA mutation and immune response in the patient. On the other hand, the possibility of using adult stem cell populations assures more abundant, accessible, and rich cell sources than embryonic/induced pluripotent stem cells or tissue-specific cells. In particular, adipose tissue has been recognized as the ideal source for stem population extraction in terms of cell abundance and tissue accessibility. Despite having already been investigated for cell therapy applications [49], adipose-derived stem cells are still little considered for Haemophilia A treatment [20,38].

This work investigated the endothelial induction of an Ad-MSC primary cell line, with the final aim to demonstrate their capability to secrete FVIII after specific stimulation. To the best of our knowledge, this is the first report about adipose stem cell capacity to produce FVIII in vitro after endothelial stimulation and without resorting to gene transfection. Based on this preliminary in vitro study, Ad-MSCs will be tested for the transplantation into the liver circulation of HA rat models in order to correct the pathologic phenotype.

As already demonstrated in the literature, stem cells can respond to specific stimuli, such as tissue damage, by migrating from their niche and entering the bloodstream to reach the site where they are recruited for tissue regeneration (homing) [50]. In the case of cell therapy strategies, cell migration from the site of the implant to the damaged tissue is a fundamental step of the regenerative process. Herein, the response of Ad-MSC to endothelial growth factors was assessed, demonstrating increased migration/chemotactic activity when the cells were conditioned by an endothelial milieu. In particular, soluble factors which were used to stimulate Ad-MSCs are proangiogenic mediators which regulate the proliferation and migration of endothelial cells and promote angiogenesis [51,52]. Since these microenvironmental cues may be present in the vascular niche associated with the endothelial sinusoid compartment of the liver, it is tempting to hypothesize an instructive effect on Ad-MSC recruitment during local and systemic administration of the cell therapy.

The differentiative potential of adipose-derived MSCs toward the endothelial lineage has already been widely demonstrated by a variety of studies focused on promoting the angiogenesis of injured tissues by stem cell-based therapy to develop new endothelial cells [35,36,37]. On the contrary, very few investigations have thought to take advantage of this property to contribute to FVIII production and restore the endothelial cell component that is not functional in Haemophilia A patients [20,38]. In this study, the acquisition of endothelial-like characteristics by induced Ad-MSCs was demonstrated at the gene and protein levels. Both early and late endothelial markers were taken into consideration. In particular, *CD34* and *PDGFRα* are generally regarded as vascular endothelial progenitor markers [53,54], while *VE-cadherin*, *CD31*, and *vWF* are expressed by mature endothelial cells [55]. As evidenced by the qPCR results, in most cases, the expression of the target genes and differentiation period were directly proportional. Only *CD31* expression was up-regulated by endothelial induction and seemed to be maintained at the same level for all the stimulation period. In parallel, flow cytometry confirmed the acquired expression of CD34, CD31, and VE-cadherin in endothelial-induced Ad-MSCs, also highlighting the positivity of stimulated cultures to VEGFR2, which was indicated as a marker of functional endothelial precursors [56].

A further element pointing to stem cell therapy for Haemophilia A treatment is the fact that a modest increase in coagulation factor expression levels is enough to obtain a healthy phenotype [57]. In this in vitro study, Ad-MSCs were confirmed to express the FVIII gene at lower levels in the undifferentiated state, demonstrating an ability to increase gene expression along time in response to the specific stimuli provided by the endothelial microenvironment. In addition to FVIII mRNA expression, it was fundamental to demonstrate protein production and secretion within the cell culture medium. The paracrine activity of undifferentiated Ad-MSCs was largely documented in the literature, their secretome being enriched with a variety of growth factors [i.e., hepatocyte growth factor (HGF), granulocyte and macrophage colony-stimulating factors, tumor necrosis factor-α (TNF-α), VEGF, brain-derived neurotrophic factor (BDNF), nerve growth factor (NGF), interleukins (ILs) 6, 7, 8 and 11], as well as extracellular vesicles [58,59]. This secretome is responsible for the trophic effects exerted by Ad-MSCs on the protection, survival, and differentiation of several endogenous cells/tissues, in addition to their immunomodulatory properties [59]. On the other hand, no studies investigated the secretome of these cells after endothelial differentiation, but it is likely to speculate that this kind of stimulation may induce an increase in the secretion of molecules/factors related to angiogenesis processes. Regarding the release of coagulation factors for Hemophilia cell therapy, Ad-MSCs were induced to secrete factors VIII and IX by gene transfer approaches [20,33,60], whereas, to our knowledge, no evidence has been reported so far about the enhancement of coagulation factor release after endothelial differentiation of these cells. In this study, only FVIII release was assessed into differentiated cell culture media since the main focus was the possible contribution of Ad-MSCs to the correction of the coagulation factor deficiency in Hemophilia A patients. The protein expression study allowed for the detection of secreted FVIII in the culture media collected by basal and conditioned Ad-MSC cultures. Remarkably, the functionality of secreted FVIII was assessed by the aPTT test.

Together, the collected data pointed out the high responsiveness of Ad-MSCs to the endothelial microenvironment, offering preliminary directions to approach the restoration of the vascular compartment with transplanted Ad-MSCs capable of secreting FVIII. Further validation of cell homing and engraftment into the liver sinusoidal compartment will be performed by in vivo studies of Ad-MSC administration to animal models affected by Hemophilia A.

Lastly, substantial limitations of this in vitro modeling study need to be mentioned, being mainly represented by the failure to replicate the innate complexity of in vivo organ systems. For example, a multitude of soluble factors and signaling molecules mediate the effects of hepatic vascular niche on stem/progenitor cell recruitment [61]. This means that the heterogeneity of the in vivo microenvironment can hardly be reproduced ex vivo. Additionally, interactions between different cell types, cell-matrix interplay, or biochemical processes of cell turnover and metabolism can hardly be captured by in vitro models, which are still far from simulating three-dimensional microarchitecture and the compartmentation of organs [62]. Other drawbacks may regard difficulties in converting in vivo doses to in vitro concentrations, as well as problems derived from long-term in vitro culture. However, this study may be considered a valid starting point for the pre-clinical translation of Ad-MSC therapy into animal HA models.

## 4. Materials and Methods

### 4.1. In Vitro Culture of Human Adipose-Derived Mesenchymal Stem Cells (Ad-MSCs)

The primary cell cultures of human Ad-MSCs were purchased from Tebu bio (Le Perray-en-Yvelines, France) and grown in complete Human Mesenchimal Stem Cell (hMSC) Expansion Media, according to the protocol recommended by the company. Briefly, the vial of cells was defrosted in a 37 °C water bath with constant, moderate agitation until the ice in the ampoule was no longer visible. Under a laminar flow hood, the vial content was transferred to a sterile 15 mL tube and centrifuged at 1500 rpm for 10 min (min) after the addition of the complete culture medium (10 mL). After discarding the supernatant, the pellet was re-suspended in 5 mL of hMSC Expansion Media, seeded into a T-25 (25 cm^2^) culture flask, and incubated at 37 °C, 5% CO_2_, and 90% humidity. The cells were ready to pass between 3 and 7 days after seeding and were sub-cultured at a density of 7000 cells/cm^2^. Ad-MSCs were purchased at passage two and grown until passage four/five before preparing the following experiments for specific differentiation.

### 4.2. Morphological Evaluation by Optical Microscopy

Ad-MSC cultures were observed daily by optical microscope DM/IL (Leica, Wetzlar, Germany) to estimate cell viability and investigate their specific morphology at different sub-confluence stages. Pictures were taken with a Nikon Digital Sight Ds-SMCc camera (Nikon Corporation, Tokyo, Japan).

### 4.3. Immunophenotype Characterization by Flow Cytometry

After expansive culture, the cells were harvested by a treatment with EDTA/tripsyn, centrifuged at 1500 rpm for 5 min and re-suspended in Phosphate-Buffered Saline (PBS) (Sigma Aldrich, Darmstadt, Germany) and 0.2% Bovine Serum Albumin (BSA) was added (Sigma-Aldrich). The cells were then labeled for 15 min at room temperature (RT) in the dark with 5 μL of the following primary antibodies: Fluorescein isothiocyanate (FITC)-conjugated anti-CD14, anti-CD45, and anti-CD105 (Santa Cruz Biotechnologies, Dallas, TX, USA); Phycoerythrin (PE)-conjugated anti-CD29, anti-CD44 and anti-CD90 (BioLegend, San Diego, CA, USA). The negative controls were prepared by labeling cells with 5 μL of FITC- and PE-conjugated isotypic antibodies (Santa Cruz and BioLegend) for 15 min at RT in the dark. The data for 10,000 total events were acquired by a FACS Canto cytometer (Becton Dickinson, Franklin Lakes, NJ, USA) and analyzed by Flowing Software 2 Turku Bioscience Centre, Turku, Finland); the results were expressed as a percentage of positive cells compared to the negative isotype control.

### 4.4. Endothelial Differentiation

For induction of endothelial differentiation, Ad-MSCs at passage four were seeded on 6-well plates at a density of 10,000 cells/cm^2^. Upon reaching about 70–80% confluence, the cells were treated for specific endothelial induction. To this end, Ad-MSCs were grown for 7, 14, or 21 days (d) in Endothelial Growth Medium (EGM)-2 (Lonza, Basel, Switzerland) with SingleQuots containing vascular endothelial growth factor (VEGF), human basic fibroblast growth factor (hFGF-b), epidermal growth factor (EGF), insulin-like growth factor-1 (IGF-1), heparin, ascorbic acid, and 2% fetal bovine serum. In parallel, the undifferentiated control was prepared by treating cells with an expansion medium only.

### 4.5. Cell Migration Assay

The stimulating effect of the endothelial microenvironment on cell migration was evaluated by the Boyden chamber experiment. Ad-MSCs (5 × 10^4^ cells/cm^2^) were seeded on the upper side of a 5.0 µm pore Transwell insert (Corning Inc., Corning, NY, USA) in differentiation medium EGM-2. Inserts were placed in a 24-well plate containing an expansion medium (lower chamber). In parallel, the chemotactic effect induced by the endothelial treatment was investigated by seeding cells in the expansion medium into the upper side of the insert and by adding EGM-2 into the lower chamber. As a control, the cells were maintained in an expansion medium in both the insert and the lower chamber. After 24 h (h), Ad-MSC cultures were fixed in 4% paraformaldehyde (PFA) (Sigma-Aldrich), and the upper membrane of the insert was swabbed to remove non-migrated cells. The membrane was cut from the insert and mounted with DAPI. Ad-MSC migration was quantified by fluorescence microscopy: the number of nuclei in the lower side of the membrane was counted in five random fields per insert (magnification × 100) by using Image J Software (U. S. National Institutes of Health, Bethesda, MD, USA).

### 4.6. Acquisition of Endothelial-like Characteristics

#### 4.6.1. Gene Expression Study by qPCR

The acquisition of endothelial-like characteristics was first investigated at the mRNA level, verifying the up-regulated expression of specific markers after cell differentiation. To this end, total RNA was extracted from both differentiated cells and undifferentiated control by using Trizol^®^ Reagent (Sigma-Aldrich), according to the manufacturer’s protocol. After quantification by the Eppendorf BioPhotometer Spectrophotometer (Eppendorf, Hamburg, Germany), the isolated RNA was analyzed for the expression of CD34, Platelet-derived Growth Factor Receptor alpha (PDGFRα), Vascular Endothelial cadherin (VE-cadherin), CD31, von Willebrand Factor (vWF), and FVIII.

For relative quantification, the target gene expression for all experimental samples is expressed as an n-fold difference referred to as a base sample called the calibrator. In this study, Human Umbilical Vein Endothelial Cells (HUVECs) were considered as the calibrator sample. After stabilization in culture, total RNA from HUVECs was isolated and quantitated as previously described.

For qPCR, the total RNA (500 ng per 100 μL final reaction volume) from the experimental and calibrator samples was reverse-transcribed using random primers and Multi-Scribe Reverse Transcriptase (High-Capacity cDNA reverse transcription kit, Applied Biosystems, Foster City, CA, USA). The reaction mixture was incubated for 10 min at 25 °C and then at 37 °C for 120 min. cDNA was stored at −80 °C until use. The transcriptional levels of the above-mentioned genes were measured by means of quantitative real-time PCR (qPCR) using the relative quantification method (2^−ΔΔCt^ method) [63]. The method was validated for our experimental system by verifying that the efficiencies of amplification of the targets and the glyceraldehyde 3-phosphate dehydrogenase (GAPDH) genes were similar. TaqMan^®^ Gene Expression Assays specific for the above-mentioned endothelial markers were purchased from Applied Biosystems (Table 1).

To avoid amplifying contaminated genomic DNA, the primer pair was placed at the junction between two exons. The qPCR assay was performed using the ABI PRISM 7300 Sequence Detection system. The PCR reaction was run in a mixture (30 μL) containing 15 μL of 2× TaqMan Universal PCR Master Mix, 1.5 μL of 20× TaqMan Gene Expression assay (all reagents from Applied Biosystems), 12.5 μL of water, and 1 μL of cDNA template. Fifty cycles of amplification were performed at 95 °C (15 s) and 60 °C (1 min), and mRNA expression levels were normalized against quantified GAPDH expression for each sample.

#### 4.6.2. Protein Expression Study by Flow Cytometry

After qPCR analysis, the activation of specific endothelial markers by differentiated Ad-MSCs was also assessed by a protein expression study. The flow cytometry analysis was performed as already described in paragraph 2.3, labeling both 21-day-differentiated cells and undifferentiated cultures with the following primary antibodies: FITC-conjugated anti-CD34 and anti-CD31; PerCP-Cy™5.5 (PC 5.5)-conjugated anti-Vascular Endothelial Growth Factor Receptor (VEGFR) and anti-VE-cadherin (Cell Signaling Technology, Danvers, MA, USA).

### 4.7. FVIII Protein Study

The capacity of Ad-MSC to release FVIII after endothelial stimulation was assessed by a dot blot assay. Culture media of 7, 14, and 21 days differentiated cells and undifferentiated control were collected and preserved at −80 °C until the time of analysis.

For Dot Blot evaluation, the samples (8 µL) were spotted onto an Immobilon^®^ membrane (Merck-Millipore, Darmstadt, Germany) and allowed to stand for 90 min at RT. The membrane was then saturated for 2 h with PBS, containing 5.0% (*w*/*w*) skim milk, and challenged for 2 h with a sheep anti-human FVIII (Affinity Biologicals, Hamilton, ON, Canada) in PBS-skim milk. After washing (3 × 5 mL, for 10 min) with PBS, the membrane was incubated for 2 h with a peroxidase-conjugated donkey anti-sheep IgG (Affinity Biologicals) as the secondary antibody. The membrane was finally stained with the ECL^®^ staining kit (GE-Healthcare, Buckinghamshire, United Kingdom) using a Molecular Imager VersaDoc MP4000 instrument (BioRad, Hercules, CA, USA). For semiquantitative analysis of the protein expression, dot blot densitometry and relative dot intensity were calculated using ImageJ software and normalizing data towards the untreated sample.

### 4.8. Coagulation Assay

The presence of biologically active FVIII in Ad-MSC supernatants was determined by performing the aPTT test, which defines the partial thromboplastin time as the time it takes for a clot to form, measured in seconds.

The aPTT was determined routinely using a HemosIL aPTT SynthASil with synthetic phospholipids and silica as an activator on an Automated Coagulation Laboratory (ACL) analyzer (ACL TOP 700 and ACL TOP 500, Instrumentation Laboratory SpA, Milan, Italy). The range of normal aPTT ratios was 0.82–1.20.

The test is based on a comparison of the ability of the different test samples to correct the aPTT of a plasma totally deficient in the coagulation factor to be measured but containing normal amounts of the other factors (i.e., FVIII deficient plasma). Thus, aPTT was measured on human FVIII-deficient plasma or mixtures (1:1) of deficient plasma and cell culture medium collected from untreated Ad-MSCs (Test sample 1) and cells treated with endothelial induction factors for 7 (Test sample 2) and 14 days (Test sample 3). An anticoagulant (sodium citrate) was also added to each test sample (9:1). Results were presented as aPTT time (seconds), aPTT ratio, and FVIII activity (%).

### 4.9. Statistics

All of the data are expressed as means of at least three different experiments ± Standard Deviation (SD). Statistical significance was determined by an unpaired *t*-test. The differences were considered to be significant with *p* values of ≤0.05.

## 5. Conclusions

This preliminary in vitro study demonstrated that Ad-MSCs are conditioned by the endothelial microenvironment by enhancing their migration/chemotaxis response to specific soluble factors and upregulating specific endothelial markers at both the gene and protein level. For the first time, we demonstrated that these cells, when properly stimulated, are able to upregulate the gene and protein expression of the coagulation factor VIII without the need to resort to gene transfection strategies. This evidence makes Ad-MSCs promising candidates to be used in Haemophilia A cell therapy and lays the groundwork for the future pre-clinical investigation of Ad-MSCs for cell therapy approaches on haemophilic animal models.

## Figures and Tables

**Table 1:** **Oligonucleotides for qPCR analysis.** TaqMan^®^ Gene Expression Assays for relative gene expression analysis. **Abbreviations:** *PDGFRα*, *Platelet Derived Growth Factor Receptor Alpha*; *VE-cadherin*, *vascular endothelial cadherin*; *vWF*, *von Willebrand factor, FVIII, factor VIII*.

Target Gene	TaqMan^®^ Probe ID	Species	Amplicon Length (bp)
*CD34*	Hs02576480_m1	*Human*	63
*PDGFRα*	Hs00998026_m1	*Human*	60
*VE-cadherin*	Hs00170986_m1	*Human*	70
*CD31*	Hs01065282_m1	*Human*	67
*vWF*	Hs01109446_m1	*Human*	56
*FVIII*	Hs00252034_m1	*Human*	127

## Data Availability

Not applicable.
